# The evolving role of paramedicine educators: A scoping review

**DOI:** 10.1016/j.afjem.2025.04.001

**Published:** 2025-04-17

**Authors:** Judy Sheahan, Richelle Duffy, Charmaine Cunningham

**Affiliations:** a University of Cape Town, Department of Family, Community and Emergency Care, Division of Emergency Medicine, Cape Town, South Africa; b Northumbria University, Faculty of Health & Life Sciences, Department Nursing, Midwifery and Health, Newcastle Upon Tyne, United Kingdom; c University of Cape Town, School of Public Health, Division of Health Policy and Systems, Cape Town, South Africa

**Keywords:** Scoping review, Paramedicine, Academia, Education, Professional identity

## Abstract

**Introduction:**

Growing responsibility and changes to paramedicine and prehospital care have led to rapid developments in paramedicine education. Despite educational requirements at increasingly advanced levels, it remains unclear how academia has responded to these changes and if they're developing the requisite skills and knowledge.

**Methods:**

A scoping review was undertaken to understand the present role of paramedicine educators and how they've adapted to the evolution of paramedicine education worldwide. Data searches were performed across eight electronic databases, six paramedicine journals, grey literature, and included sources reference lists.

**Results:**

The four-staged search strategy revealed 1,738 sources, of which 32 remained for final synthesis. In general, there was a lack of contemporary research examining the role of the paramedicine educator despite changes to Higher Education provision and function of paramedics. Noteworthy was the absence of articles from Africa, South America, and major parts of Europe, highlighting the need for development in these regions. There is a lack of clear role descriptions or definitions for paramedicine educators. Inconsistencies were highlighted in entry criteria and progression routes across paramedicine academia globally, emphasising the importance of support for transitioning and established paramedicine educators.

**Discussion:**

These findings have important implications for Higher Education. Professional demands are on the rise, creating a need to introduce clearly defined roles for paramedicine educators to provide clarity in expectations and increase confidence. The changing landscape of care provision in paramedicine from the historic emergency care focussed model to a more autonomous and inclusive sphere of out-of-hospital care, provides an ideal opportunity to progress and shape the identity of the paramedicine educator.

## Highlights

• The evolution of EMS from emergency care-focused models to more community orientated and inclusive out-of-hospital care aligns with the evolving healthcare needs of African communities, presenting opportunities to shape the academic identity of EMS (paramedicine) educators.
• Establishing a consensus on academic nomenclature and standardising minimum qualifications required (both clinical and pedagogical) and recognising clinical expertise alongside academic credentials can contribute to building sustainable and appropriate EMS education programs across Africa.
• Developing a bespoke, guided transition process for EMS clinicians entering academia, including mentorship and individualised professional development, can address challenges unique to African contexts, such as the diverse scope of EMS systems and resource limitations.

## African relevance

▒

• The evolution of EMS from emergency care-focused models to more community orientated and inclusive out-of-hospital care aligns with the evolving healthcare needs of African communities, presenting opportunities to shape the academic identity of EMS (paramedicine) educators.
• Establishing a consensus on academic nomenclature and standardising minimum qualifications required (both clinical and pedagogical) and recognising clinical expertise alongside academic credentials can contribute to building sustainable and appropriate EMS education programs across Africa.
• Developing a bespoke, guided transition process for EMS clinicians entering academia, including mentorship and individualised professional development, can address challenges unique to African contexts, such as the diverse scope of EMS systems and resource limitations.

▒

## Introduction

Paramedicine has evolved from vocational education training (VET) to formal harmonised curriculum-based frameworks of education across many developed countries [[1], [2], [3]]. Australia, the United Kingdom, and South Africa introduced their first university-level paramedic education in 1995, 1998, and 2000 respectively, with further educational developments leading to the introduction of masters and doctoral paramedicine programmes [4]. Despite the global evolution of education and professional standards for paramedicine, there are no agreed standards or definitions in place for paramedicine educators. Inconsistencies are evidenced by varying terminology used in academic paramedicine literature, inconsistencies in job descriptions between educational institutions in the same countries, and variable requirements to fill academic positions [5,6]. Recruitment criteria often prefer clinical qualifications and experiential knowledge over academic expertise [7].

Within general Higher Education (HE), various academic titles are evident, including trainer, instructor, educator, lecturer, junior lecturer, associate lecturer, senior lecturer, associate professor, and professor [8]. Evidence suggests that titles awarded to persons in academia directly impact their performance and subsequent professional outputs [9,10]. Clarity in the description of titles and their relation within and outside educational organizations are linked to personal and professional satisfaction, local and international skill transferability and streamlined career progression [11,12]. Assimilation with one's job title or role description plays a significant role in the transformation and evolution of professional identity [13], and having an established professional identity directly impacts one's confidence and the quality of work [11,12].

It is therefore helpful to make the distinction between the terms academic and educator. Generally, academics are educated to doctoral level, active researchers in their discipline, and producing research outputs [14,15]. Educators are primarily focus on teaching and facilitating learning. Even with these distinctions, the terms are used interchangeably within research leading to ambiguity within the individual about who is awarded which title [8]. Current literature remains unclear in application of the terms academic and educator. While some institutions do clarify the terms and academic nomenclature, it remains inconsistent between institutions per nation and globally [16].

This scoping review explored the role description of paramedicine educators to answer the research question: *What do academia, and the roles of educator and academic mean in paramedicine?* The findings can be used to help paramedicine educators identify and frame their professional identity early in their careers [17,18], subsequently translating to improved confidence in academic and pedagogical behaviours and conduct [19].

## Methods

The study followed the JBI methodology for scoping reviews [20]. Reported data adhered to the preferred reporting items for systematic reviews and meta-analysis extension for scoping reviews (PRISMA-ScR) [21]. The protocol was registered with Open Science Framework – available at https://osf.io/3jk6f/ [22].

### Eligibility criteria

Inclusion and exclusion criteria are shown in Table 1.

**Table 1:** Inclusion and exclusion criteria.

Inclusion criteria	Exclusion criteria
Publication date post-1990	Publication before 1990
Publication in English, or translation to English available for free	Publication not in the English language
Full-text availability Peer reviewed	Context of study not related to paramedicine or paramedicine academia
International literature All types of literature, including grey literature	

### Data sources and search strategy

Table 2 depicts the data sources used in the search strategy undertaken between July 2023 and April 2024. Reference lists of all included articles were searched for additional citations. Electronic databases was searched using a combinations of terms (Table 3) and the Boolean search operators ‘AND’/‘OR’ across journals dedicated to paramedicine. Grey literature included conference proceedings, theses, dissertations and HE online repositories. Appendix 1 provides a summary of the CINAHL database search.

**Table 2:** Data sources.

Eight electronic databases	Six Journals hand-searched	Grey literature
Medline	Journal of Paramedic Practice	Google
CINAHL	Australasian Journal of Paramedicine	Research Gate
Africa-Wide Information	British Paramedic Journal	
Education Database	South African Journal of Pre-hospital Emergency Care	
ERIC	Prehospital Emergency Care International Paramedic Practice	
Scopus		
Web of Science		
Google Scholar

**Table 3:** Boolean search operators.

AND	emergency medical services, EMS, ambulance service, ambulance, paramedicine, paramedic ‘AND’ academia, academic, lecturer, educator, teacher
OR	educator, academic, academia

### Study selection and screening

The initial searches were conducted in Medline and CINAHL with the assistance of an affiliated librarian, revealing 103 results. Title and abstract screening were conducted, resulting in 93 articles being excluded. The inclusion of additional search terms was deliberated by the research team and deemed unnecessary. Ten articles remained for full-text review. The search strategy and selection criteria were applied to all remaining databases, resulting in an additional 746 search results. After title and abstract screening, duplicates and one non-English article were removed, with 21 articles remaining for full-text review. Hand-searching the journals resulted in 878 results, with four being included for full review. Six articles were identified for full review from reference lists, and grey literature searches contributed five articles; totalling the number of articles for full review to 46. After a full review, 14 articles were excluded (Appendix 2 provides details). Full-text reviews were initially conducted by the lead author, after which it was reviewed independently by co-authors, no disagreements for final inclusion required resolution.

### Data extraction and synthesis

Initial data extraction was conducted by the lead author and captured in an Excel spreadsheet. Items charted included authors, publication year, country of origin, purpose of study, population and sample size, methodology, findings, and limitations. It was reviewed by the co-authors to increase inter-rater consistency [23]. Variations in charting or interpretation of charted data were resolved by consensus discussion amongst the research team. Articles included in the final synthesis were read and systematically analysed through a descriptive qualitative lens. These were independently contextualized to the research question by each author and then compared, discussed and agreed upon.

## Results

**Fig. 1:**
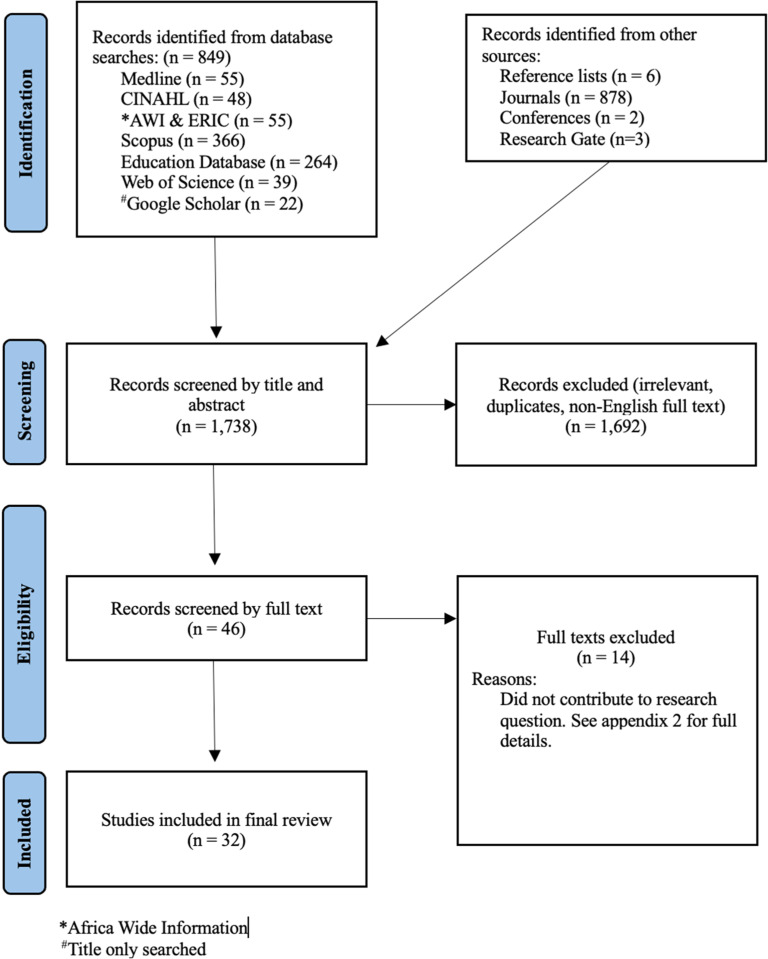
PRISMA flow diagram [24].

The four-staged search strategy revealed 1,738 sources, of which 32 remained for final synthesis. The results of the search strategy and study selection process are shown in Fig. 1. Appendix 3 provides details of the included articles and charted data. Data is presented by general characteristics and four emerging themes.

### General characteristics of included articles

**Fig. 2:**
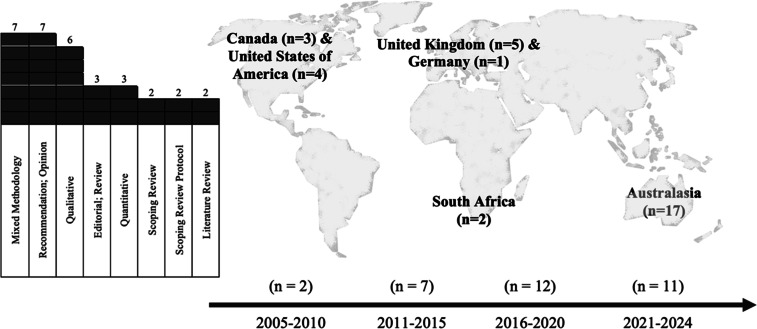
Types of research; Geographical origin; and publication dates.

Fig. 2 depicts the types of research methodologies, the geographical origin of included articles, publication dates. There was a noted lack of contemporary research examining the role of the paramedicine educator despite changes to HE provisions and the role and function of paramedics. There were no articles included from South America, most of Europe, and mid to Northern Africa. While included articles represent predominantly high-income counties (HIC), other nations are represented, making the findings applicable to broader paramedicine education.

### Lack of clarity in terms used to describe paramedicine educators

Throughout the literature different terms such as academic, lecturer, educator, teacher, or faculty are used within *single* articles to refer to the same people groups [5,6,[25], [26], [27], [28], [29], [30], [31], [32], [33], [34], [35]]. Further there were contradicting explanations for paramedicine academics from within the same regions [6,[36], [37], [38]], and no consistency in using academic terms globally. Described for the first time in 2006 and multiple times since, a paramedicine academic is considered someone who holds at least a doctoral qualification and publication record [37,[39], [40], [41]]. In the Australasian region, it is said without these credentials full-time appointment in HE is unlikely. In 2018, the academics repertoire was expanded to include teaching clinical knowledge and skills, in addition to being an active research scholar [38]. The first variance originated in the same year when a paramedicine academic was described only as holding a doctoral qualification and publication record, and an educator/lecturer someone who teaches. The educator/lecturer was clearly pronounced *not an academic* due to lack of doctoral qualification [37]. In Europe, distinction is made between instructor and educator. An instructor is a paramedic specialized in on-the-job training (with no elaboration), and an educator is a paramedic no longer clinically active that lectures, practice exercises and simulations [42]. A recent publication adds that the current paramedicine academic workforce is junior and lacks post-doctoral academic leadership [29]. The same article describes 161 persons as academics, yet only 29 hold doctoral qualifications, contradicting previous descriptions of academics in the same region.

In Canada, an educator is one of many roles that paramedics occupy in their day-to-day practice. Initially described as ‘patient educator’, and then as an educator supporting opportunities in their own practice, such as self-development and peer learning [43]. In the same article, educators along with researchers and policy makers are recommended as key stakeholders responsible for analyses and validation of study findings. Lastly, an educator is the end-user of validated results to inform curriculum development and assessment strategies. Here, the single term ‘educator’ was used in distinctly different settings of paramedicine, which is confusing, as each settings requires unique skill sets.

In the last decade, roles such as research paramedic and paramedic lecturer practitioner emerged [[44], [45], [46]]. These terms result in unclear role descriptions that intersect both the clinical and academic environments. While in principle it could be seen as progressive for the profession [47], it merely adds to the already ill-defined academic terminology. Terms used to describe individuals in paramedicine academia are either interchangeable or distinctly separated, each carrying different meanings in the academic context; therefore, they should be used appropriately [32]. HE is considered the agents of professionalisation, informing research agendas and future practice; however, there is no consensus on their role in this [31,48].

### Challenges faced when transitioning into HE and readiness for the role

Many paramedics who made the transition into HE described the process as not what they expected, feeling lost, alone and confused [18,37]. The transition to HE was without clear expectations, limited guidance, induction or mentorship. Pedagogical knowledge was usually assumed, paralleled with the immediate pressures to do research, publish, and pursue doctoral qualifications [18,40]. Sessional or part-time work was considered to provide a more scaffolded transition into HE, yet part-time paramedicine educators reported feeling disconnected from their full-time colleagues [49]. Additionally, the ad hoc recruitment nature of part-time work places less emphasis on merit, and advantages those who are familiar with colleagues already in HE [5].

Having a doctoral qualification upon entry to academia did not ease the burden of lacking pedagogical skills. Entry level academics (with or without doctoral qualifications) perceived the emphasis on delivering good quality education inferior to research outputs [40]. It was made clear that paramedicine educators are underprepared to be academics, and in some regions paramedicine remains perceived as vocation rather than a profession [50]. Educators were described as “*...responsible for teaching the greatest number of didactic and skills lab hours...”* [51]. From this description, an inference can be drawn that an educator is associated with teaching, and teaching methods used are outdated. This sentiment of lack in pedagogical skills, or support to develop pedagogical skills was echoed multiple times [6,18,38,41].

### Balancing the multiple demands of research, scholarship, and pedagogy

Many paramedicine educators found it challenging to balance the desire to obtain pedagogical knowledge and improve student-centred responsibilities with research outputs and obtaining doctoral qualifications [18,27,40,51]. Paramedicine educators reported feeling conflicted about putting the needs of students first, or focussing on establishing their own academic status [40]. Early career academics are confused; assumingly employed based on their clinical expertise, now expected to perform pedagogical tasks, research, administration, and support students with limited support in any of these domains [27]. Paramedicine educators frequently stated that high quality teaching and learning was overshadowed by the emphasis on obtaining research grants and publications [27,40]. Noteworthy that paramedic students responded more positively towards educators who had some formal HE credentials and rated them higher [52]. Paramedicine educators recommended that institutional support and collaboration with senior colleagues can play a crucial role in managing demands, promoting a sustainable and fulfilling academic career [29,49,53].

### Role satisfaction, collaboration opportunities and lack of career pathways

There was dissatisfaction with the work environment with reports of being overworked and unsupported [50,51]. The aforementioned nature of part-time appointment with feeling disconnected from full-time educators was an identified barrier to role satisfaction [5]. Progression pathways in academia is inconsistent, resulting in unclear future opportunities for paramedicine educators [5,38]. The possibility of dual academic-clinician roles is uncertain likely due to the lack of role descriptions and collaboration requirements [44,45,54]. Appointment of paramedicine graduates into academia without clinical experience places questionable value on the requirement and transferability of experiential knowledge [47]. With the lack of collaboration between the clinical and educational environments creating barriers in preparing clinicians as educators, and contrariwise, academia hinders continued clinical development in educators [29,39]. There appears to be the imposed notion of choice: paramedicine clinician or paramedicine educator [37].

Paramedicine literature highlights concern about the importance of and support for ongoing academic professional development [27,34,44,49,51,53]. The profession universally has undergone changes from VET to HE, yet there are no explicit development plans to support educators through it [53,54]. The neo-liberal stance on HE prioritises student satisfaction, neglecting staff needs [5,27]. Paramedicine educators have highlighted the lack of pedagogical performance, limited opportunities for targeted professional development, and absent educational leadership [5,29,49].

### Strengths and limitations

This scoping review provides a current perspective on the descriptions of paramedicine educators, and the results can inform the premise towards establishing universal paramedicine academia vocabulary. This review was comprehensive and included all types of research, but relevant studies may have been excluded due to the limitation of English publications. Most articles in the review were from HIC countries, thus results may not be directly relatable to low-middle income countries (LMIC) due to differences in paramedicine HE and associated resource constraints. In addition, 10 of the 17 articles from the Australasian region were published by one or more of the same three authors. Although esteemed paramedicine academics, this inherently provides a less generalisable perspective. Finally, differences in pathways to obtaining paramedic status, regulatory authorities and/or professional bodies governing paramedicine academia can impact the application of results globally.

## Discussion

This scoping review considered the role description of paramedicine educators. The 32 articles included in the final synthesis provide varying descriptions of paramedicine educators, but a core understanding of what it means to be a paramedicine academic remains elusive. The transition into HE is challenging, and paramedicine educators are underprepared for academic roles adding to poor role satisfaction, the inability to effectively collaborate and uncertainty in career progression pathways. The transition into paramedicine education involves a significant shift in professional identity [55]. Identity formation and transformation is a dynamic process influenced by socialization, mentorship and experiential learning [56]. This scoping review highlights key challenges related to professional identity, including struggles of academic legitimacy due to lack of formal HE education and academic job expectations. The findings suggest that structured mentorship and institutional support play a crucial role in facilitating this transition, reinforcing the importance of professional development in shaping thriving paramedicine educators.

The titles given to paramedicine educators vary greatly across findings, including a range from junior lecturer to professor, causing inequality about expectations and reward across different institutions [37]. Whilst the use of titles may seem like a trivial concept, it has been found to impact assimilation with role description and plays a role in the transformation and evolution of professional identity [32]. This has implications for self-confidence and the quality of work in health education [[9], [10], [11], [12], [13]].

With inconsistent entry criteria and progression in paramedicine academia globally ranging from clinical qualifications and experiential knowledge to doctoral qualifications, this review exposed that upon entry to paramedicine academia, many academics feel unprepared and unsupported [18,38,40,48,50,51]. The evident lack of collaboration between clinical and academic environments, combined with limited transition pathways from clinical to academic careers resulted in working environments being ‘not as expected’. Thus, many educators revert to remaining in the clinical environment where they are more comfortable and have the required knowledge to perform effectively [[37], [38], [39],^53^].

This can in part be attributed to academic hegemony and the growing demands placed upon academics by neo-liberal economic policies transforming teaching, learning, and research. However, paramedicine educators lack the knowledge and experience to effectively respond when moving from clinical practice creating unequal power relations and further fuelling inequities [5,18,29,38,39]. Whilst some reference is made to introductory sessions for new academic staff, these are not ongoing and lack context to paramedicine [40,49].

Paramedicine educators perceived being viewed as *non-academic* [15,18,36,37]. It is well established that working in academia requires a broad range of responsibilities, all of which require a unique set of knowledge and skills. The findings indicate that a doctoral qualification upon entry to academia does not translate to adequate pedagogical knowledge [40], whilst simultaneously formal teaching qualifications may not adequately prepare for research and publications. No data reported the number of paramedicine academics with formal teaching qualifications.

## Conclusion

In paramedicine HE, the shift from a historic emergency care-focused model to a more autonomous and inclusive out-of-hospital care approach aligns with global community needs [57]. This change presents an ideal opportunity to advance and shape the academic identity of the paramedicine educator. This review highlights the need for global standards and consensus regarding paramedicine academic nomenclature and qualifications. Furthermore, entry routes to academia should include guided transition processes with academic mentorship and personalised support. These processes can alleviate the transition challenges experienced by early career academics creating a solid foundation upon which balancing the multiple demands of academia can be developed. It is recommended that career progression encourages professional development based on individualised interest, bespoke and structured training programs for developing pedagogical skills and improving integration between clinical and academic roles. The increased focus on the student experience and lifelong learning providing the impetus to challenge the current status quo [58].

We recommend that the paramedicine profession aims to establish consensus on academic vocabulary. We further recommend that priority is given to research exploring factors affecting the transition and adapting to paramedicine academia specifically in LMIC where paramedicine education may differ to due to resource constraints and where current literature is lacking. This could inform future research towards a bespoke paramedicine academia transition framework, aimed at supporting entry into academia and shaping the identity of the paramedicine educator, applicable to the global paramedicine profession.

## Ethics statement

Not applicable.

## CRediT authorship contribution statement

**Judy Sheahan:** Conceptualization, Methodology, Project administration, Investigation, Formal analysis, Visualization, Writing – original draft. **Richelle Duffy:** Supervision, Formal analysis, Validation, Writing – review & editing. **Charmaine Cunningham:** Supervision, Formal analysis, Validation, Writing – review & editing.

## Declaration of competing interest

The authors declare that they have no known competing financial interests or personal relationships that could have appeared to influence the work reported in this paper.

The author CC is an Associate Editor for this journal and was not involved in the editorial review or the decision to publish this article.
